# P16. Differential susceptibility of human and mouse NK cells to malignant cell-induced abnormalities in autologous combinations: a potential mechanism for the NK cell-based immunotherapy efficacy

**DOI:** 10.1186/2051-1426-2-S2-P7

**Published:** 2014-03-12

**Authors:** G Sconocchia, R Arriga, S Caratelli, A Coppola, GC Spagnoli, G Lanzilli, B Capuani, F Ferrelli, D Lauro, S Ferrone

**Affiliations:** 1 Translational Pharmacology CNR, Biomedicine, Rome, Italy; 2 University of Rome Tor Vergata, Systems Medicine, Rome, Italy; 3 Surgical Research and Hospital Management, University of Basel, Biomedicine, Basel, Switzerland; 4 Massachusetts General Hospital, Harvard Medical School, Surgery, Boston, MA, USA

## Background

Natural killer (NK) cells are highly effective in controlling tumour growth, in mice, but have no significant effect in humans. The reason(s) of this phenomenon is(are) unclear.

## Methods

The effects of cancer cells on NK cells during target-effector cell conjugation was investigated utilising standard immunological methods including flow cytometry, chromium release and enzyme-linked immunosorbent assays while gene expression was evaluated by quantitative reverse transcriptase-polymerase chain reaction.

## Results

We found that this phenomenon was associated with the different susceptibility of human and mouse NK cells to autologous tumour cell-induced NK cell abnormalities (NKCA). The latter includes CD16 down-regulation and NK cell depletion. Induction of NKCA by leukaemia and solid tumour cells was influenced neither by IL2 treatment nor by HLA class I antigen expression, but was abrogated by a 10 day culture. Following a 10 day of PBMCs culture, NK cells became resistant to leukaemia and solid tumor cell induced NKCA but maintained their cytotoxic activity. Actinomycin D restored the susceptibility of long term NK (LTNK) cells to NKCA suggesting that the generation of resistance to NKCA required RNA transcription. TAPI-0, a functional analogue of the tissue inhibitor of metalloproteinases (TIMP) 3 inhibited cancer cell induced NKCA underlying a role for a restricted number of metalloproteinases in the generation of this phenomenon. Finally, we found an association of TIMP3 gene and protein over-expression with the reduced susceptibility of LTNK cells to cancer cell induced NKCA.

## Conclusions

This study provides evidence that TIMP3 plays a role in the protection of LTNK cells from cancer cell induced NKCA.

